# Chromatin dynamics enable transcriptional rhythms in the cnidarian *Nematostella vectensis*

**DOI:** 10.1371/journal.pgen.1008397

**Published:** 2019-11-06

**Authors:** Eviatar N. Weizman, Miriam Tannenbaum, Ann M. Tarrant, Ofir Hakim, Oren Levy

**Affiliations:** 1 The Mina & Everard Goodman Faculty of Life Sciences, Bar-Ilan University, Ramat-Gan, Israel; 2 Department of Biology, Woods Hole Oceanographic Institution, Woods Hole, Massachusetts, United States of America; MRC Clinical Sciences Centre, UNITED KINGDOM

## Abstract

In animals, circadian rhythms are driven by oscillations in transcription, translation, and proteasomal degradation of highly conserved genes, resulting in diel cycles in the expression of numerous clock-regulated genes. Transcription is largely regulated through the binding of transcription factors to *cis-*regulatory elements within accessible regions of the chromatin. Chromatin remodeling is linked to circadian regulation in mammals, but it is unknown whether cycles in chromatin accessibility are a general feature of clock-regulated genes throughout evolution. To assess this, we applied an ATAC-seq approach using *Nematostella vectensis*, grown under two separate light regimes (light:dark (LD) and constant darkness (DD)). Based on previously identified *N*. *vectensis* circadian genes, our results show the coupling of chromatin accessibility and circadian transcription rhythmicity under LD conditions. Out of 180 known circadian genes, we were able to list 139 gene promoters that were highly accessible compared to common promoters. Furthermore, under LD conditions, we identified 259 active enhancers as opposed to 333 active enhancers under DD conditions, with 171 enhancers shared between the two treatments. The development of a highly reproducible ATAC-seq protocol integrated with published RNA-seq and ChIP-seq databases revealed the enrichment of transcription factor binding sites (such as *C/EBP*, homeobox, and *MYB)*, which have not been previously associated with circadian signaling in cnidarians. These results provide new insight into the regulation of cnidarian circadian machinery. Broadly speaking, this supports the notion that the association between chromatin remodeling and circadian regulation arose early in animal evolution as reflected in this non-bilaterian lineage.

## Author summary

The phenotype of an organism cannot be fully explained by its genome; it is now clear that other factors contribute to the ecology and evolution of animals. The DNA molecule is exceptionally long and has to fit into a small nucleus; therefore, the DNA is wound around proteins called histones in a unique architectural complex called a nucleosome (that further forms the chromatin). This DNA structure plays a crucial role in gene regulation, as nucleosomes alternate accessibility to regulatory sites, that in turn navigate expression patterns that allow gene expression or suppression. It is well known that chromatin remodeling is associated with circadian regulation in mammals, but it was uncertain whether cycles in chromatin accessibility are a conserved feature of clock-regulated genes throughout the animal kingdom. We used ATAC-seq and RNA-seq data to show that the association between chromatin remodeling and circadian regulation arose early in animal evolution as reflected in the sea anemone, *Nematostella vectensis*, model organism.

## Introduction

Circadian clocks are present in most organisms and have evolved to help organisms anticipate daily and seasonal rhythms and adjust their biochemical, physiological, and behavioral processes accordingly. The molecular basis of the endogenous clock apparatus is manifested by transcriptional machinery that is controlled by regulatory factors and organized in auto-regulatory feedback loops [1]. In mammals, these temporal oscillations in gene expression are paralleled by genome-wide chromatin remodeling events that provide flexibility to circadian regulation [2]. In mammals, several chromatin remodelers are involved in circadian regulation, including the core circadian protein CLOCK, which can operate as an acetyltransferase on histone H3 at K9 and K14; this acetylation is associated with a permissive chromatin state for transcription [3]. The CLOCK histone acetyltransferase (HAT) domain was previously shown to be conserved across species [4,5], and similar mechanisms of the circadian epigenome have been suggested in *Drosophila* [6,7]. However, no study to date has investigated chromatin dynamics concerning the biological clock in non-bilaterian animals.

Eukaryotic DNA is wound around histone proteins in a complex called a nucleosome, which helps to compress the molecule into the cell nucleus. This complex is regulated as histones are removed to expose regulatory sites, such as *cis*-regulatory elements (CREs) and promoters, to allow the binding of transcription factors (TFs) and other regulatory proteins. Identification of enriched motifs with these active CREs can, therefore, reveal genes associated with the regulation of the transcriptional network [8]. Genome-wide mapping of TFs binding to chromatin is frequently done by chromatin immunoprecipitation (ChIP) based methods, such as ChIP-seq [9]. However, these techniques are expensive and require a significant amount of tissue and extensive processing of the sample. An assay for Transposase-Accessible Chromatin with high-throughput sequencing (ATAC-seq) is a technology that favors the sequencing of accessible chromatin loci [10] and has the potential to overcome these limitations. While ATAC-seq is a powerful and promising approach for epigenetic regulation research, it has primarily been applied within well-characterized model systems.

In this study, we set out to expand the current knowledge of metazoan circadian gene expression regulation by understanding the interplay between chromatin accessibility and circadian gene expression dynamics. We focused on the phylum Cnidaria, the sister-lineage to bilaterian animals, and specifically chose the sea anemone, *Nematostella vectensis*, which has emerged as a model for studying development, differentiation, and more recently, circadian regulation [11,12]. *N*. *vectensis* is widely distributed in shallow brackish environments and unsurpassed regarding the ease with which its entire lifecycle can be maintained in the laboratory [13,14]. Studies of *N*. *vectensis* locomotor activity and rhythmic gene expression, including previous work by our group [15–18], have provided a first glance into the evolution of the metazoan circadian clock (S1 Fig). In this study, developing an optimized protocol and applying ATAC-seq enabled us to detect accessible chromatin regions under light-dark (LD) and constant darkness (DD) conditions, and refine our lab’s previous findings [15]. These findings led us to hypothesize that circadian modulation of chromatin remodeling occurs on a greater scale than previously shown by gene expression profile only. Integrating chromatin accessibility profiles with transcription profiles (RNA-seq) revealed that the majority of cyclic genes were associated with ATAC-seq peaks within their promoters. This work opens a path into the evolution of basal metazoans’ circadian transcription and regulation, showing the association of gene activity with chromatin accessibility. Therefore, chromatin structure may play an important role in regulating circadian gene expression in *N*. *vectensis*.

## Results

### Nuclear isolation and ATAC-seq analysis from whole animal samples

**Fig 1:**
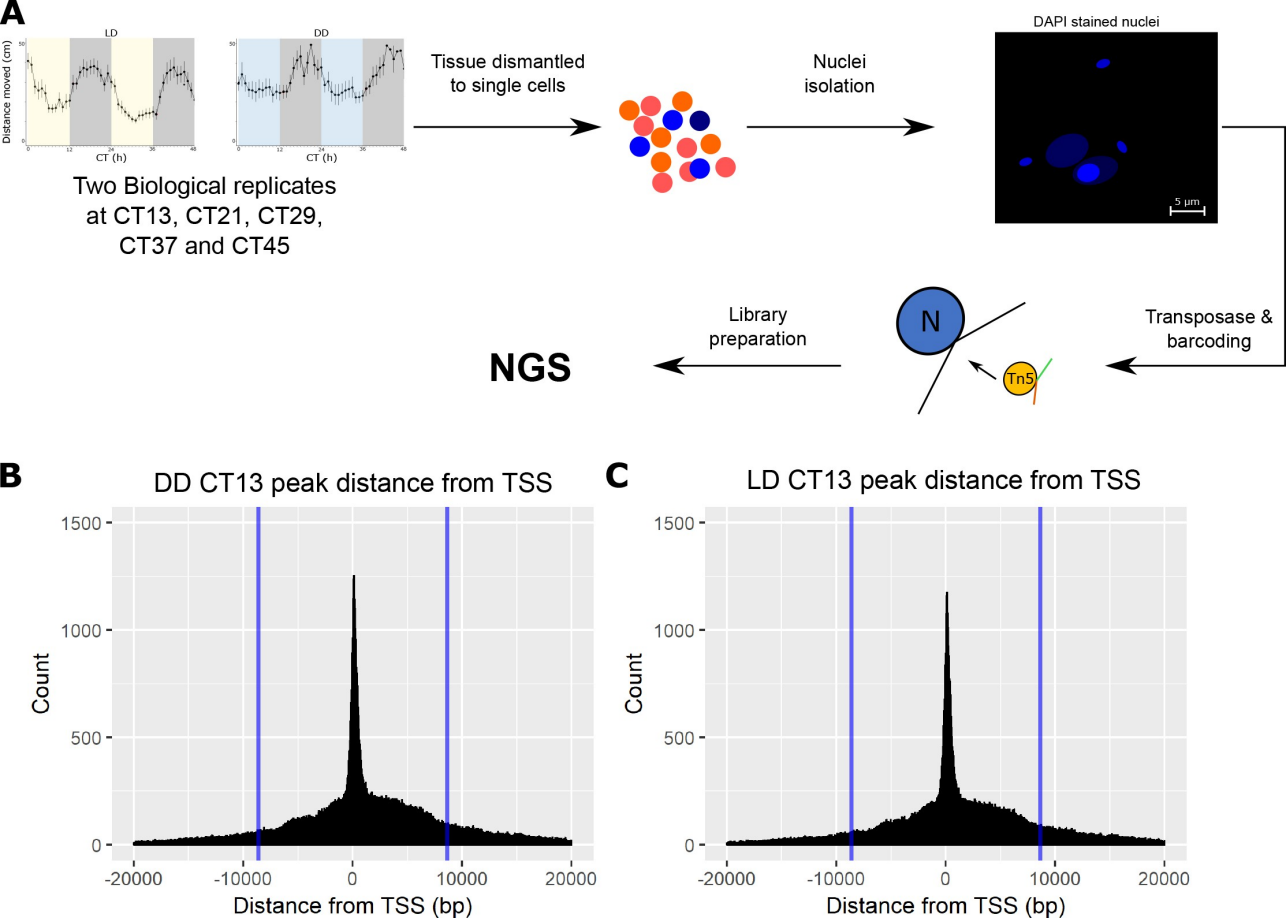
(A) A scheme describing the ATAC-seq process from sampling of whole animals to ATAC-seq analysis. (B and C) Example histograms from CT13 LD and DD, showing the peak distribution around TSS. The blue bars indicate the approximate place of the next nearest TSS (~8,600bp).

ATAC-seq was applied to measure high-resolution chromatin accessibility in *N*. *vectensis* under two light regimes, resembling maximum and minimum temporal behavioural activity. Nuclear integrity [19] was achieved by (i) dissolving animal tissue into single cell suspensions and (ii) separating released nuclei from other organelles and cytoplasmic debris to reduce non-nuclear DNA contamination, as presented in Fig 1A. Each ATAC-seq library was prepared using a transposition reaction from ~400,000 cells that were sampled from one individual animal, from circadian time 13 (CT13) to circadian time 45 (CT45), in 8 hour intervals. Libraries were sequenced from two independent biological replicates from the LD treatment and compared to two independent biological replicates collected at the same time, under DD. After filtering the PCR duplicates, generated from the library amplification process and irregular reads between the biological replicates, our ATAC-seq libraries showed a median depth of 10 million unique, high-quality mapped reads per sample (Tables 1, 2 and Fig 1). As presented in S1 Table, the biological replicates were highly similar (average R^2^ = 0.96 SD ±0.014 and p-value<0.01), demonstrating highly reproducible data from *N*.*vectensis* whole animal sampling. Furthermore, the significant peaks (8366–36582 peaks with an irreproducible discovery rate (IDR) cut-off of 0.05) from both LD and DD treatments were clustered around transcriptional start sites (TSSs, Fig 1B and 1C and S2 Fig).

**Table 1:** Alignment rate to *Nematostella vectensis* genome (Genome version: Nemvec1).

Sample name	total reads (abs)	aligned 0 times (%)	aligned exactly 1 time (%)	aligned >1 times (%)	overall alignment rate (%)	number of reads after filtering (abs)
CT13 DD sample 1	59,029,685	7.77	34.45	57.78	92.23	14,507,719
CT13 DD sample 2	55,841,194	6.93	33.51	59.57	93.07	12,704,274
CT13 LD sample 1	54,995,451	4.51	29.66	65.83	95.49	12,718,720
CT13 LD sample 2	52,496,158	7.05	26.41	66.54	92.95	12,413,764
CT21 DD sample 1	8,294,283	21.47	30.40	48.13	78.53	3,559,809
CT21 DD sample 2	8,474,428	15.23	22.16	62.61	82.33	3,795,098
CT21 LD sample 1	12,275,217	16.57	26.87	56.56	83.43	6,159,364
CT21 LD sample 2	10,714,787	8.34	24.02	67.64	91.66	7,124,575
CT29 LD sample 1	10,940,986	7.77	25.34	66.88	92.23	7,501,810
CT29 LD sample 2	16,575,401	8.39	27.19	64.41	91.61	11,111,179
CT37 DD sample 1	42,463,524	20.34	33.45	46.21	76.61	8,955,328
CT37 DD sample 2	41,693,108	28.49	32.21	39.30	71.51	8,691,388
CT37 LD sample 1	25,195,841	16.68	29.38	53.94	83.32	7,225,885
CT37 LD sample 2	47,864,491	31.57	32.36	36.07	68.43	6,946,050
CT45 DD sample 1	45,241,651	3.53	28.46	68.01	96.47	27,004,604
CT45 DD sample 2	28,706,397	4.95	27.58	(67.47	95.05	14,626,385
CT45 LD sample 1	47,725,181	4.26	30.70	65.03	95.74	27,581,593
CT45 LD sample 2	45,396,395	5.03	31.45	63.51	94.97	25,347,195

**Table 2:** Alignment rate to *Nematostella vectensis* mitocondria sequence (Version: Nematostella sp. JVK-2006 mitochondrion).

Sample name	total reads (abs)	aligned 0 times (%)	aligned exactly 1 time (%)	aligned >1 times (%)	overall alignment rate (%)
CT13 DD sample 1	59,029,685	95.80	4.20	0.00	4.20
CT13 DD sample 2	55,841,194	96.60	3.40	0.00	3.40
CT13 LD sample 1	54,995,451	96.82	3.18	0.00	3.18
CT13 LD sample 2	52,496,158	94.71	5.29	0.00	5.29
CT21 DD sample 1	8,294,283	94.88	5.12	0.00	5.12
CT21 DD sample 2	8,474,428	95.75	4.25	0.00	4.25
CT21 LD sample 1	12,275,217	93.66	6.34	0.00	6.34
CT21 LD sample 2	10,714,787	94.77	5.23	0.00	5.23
CT29 LD sample 1	10,940,986	96.85	3.15	0.00	3.15
CT29 LD sample 2	16,575,401	96.45	3.55	0.00	3.55
CT37 DD sample 1	42,463,524	96.32	3.68	0.00	3.68
CT37 DD sample 2	41,693,108	95.05	4.95	0.00	4.95
CT37 LD sample 1	25,195,841	95.44	4.56	0.00	4.56
CT37 LD sample 2	47,864,491	93.77	6.23	0.00	6.23
CT45 DD sample 1	45,241,651	96.55	3.45	0.00	3.45
CT45 DD sample 2	28,706,397	95.74	4.26	0.00	4.26
CT45 LD sample 1	47,725,181	97.75	2.25	0.00	2.25
CT45 LD sample 2	45,396,395	95.69	4.31	0.00	4.31

### ATAC-seq provides a glimpse into *N*. *vectensis* genome regulatory regions

**Fig 2:**
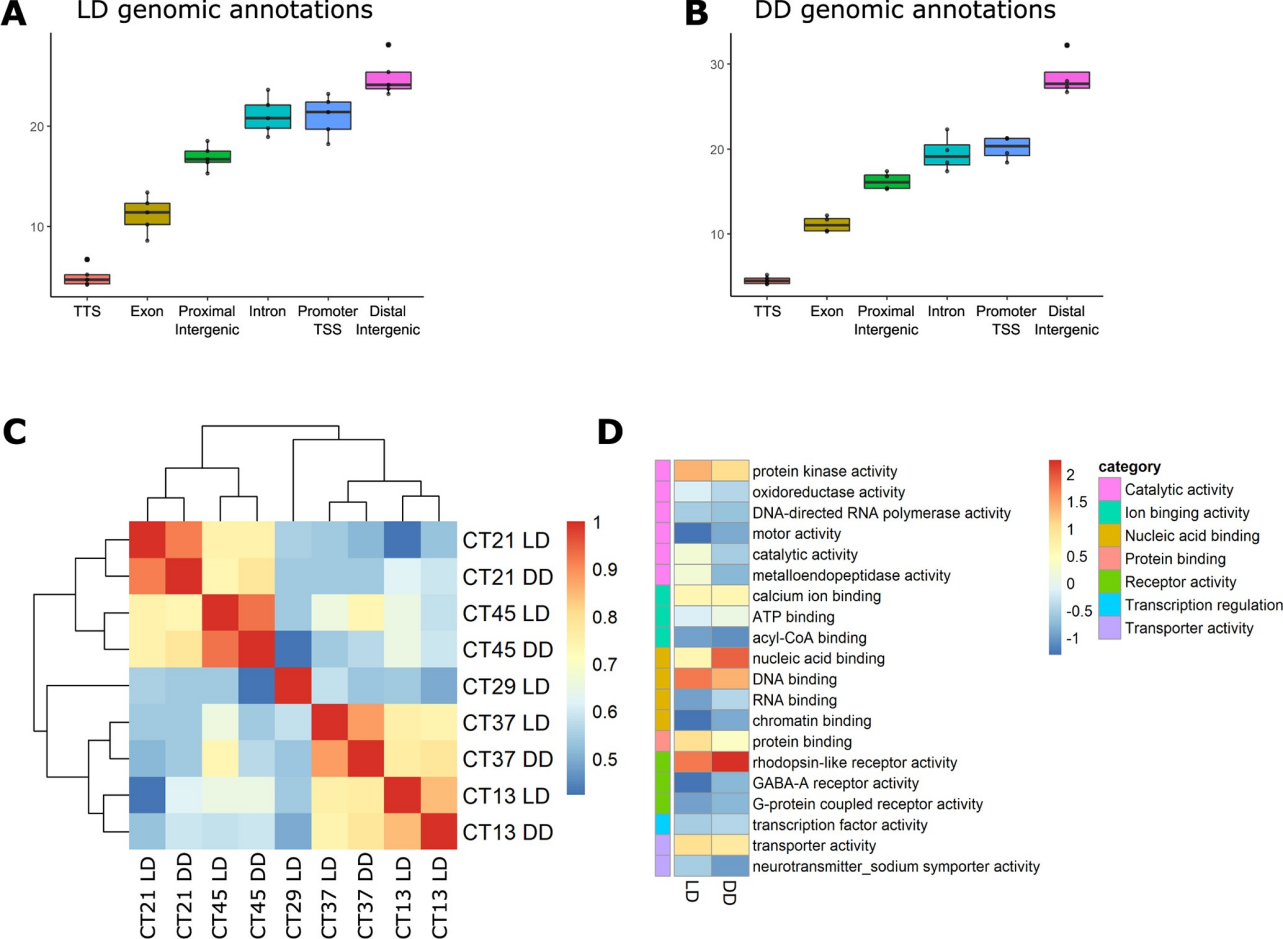
(A) Boxplot representing the percentage of genomic features of LD-treated *Nematostella vectensis*, calculated from biological replicates across experimental sampling points (n = 5). (B) Boxplot representing the percentage of genomic features of DD-treated *Nematostella vectensis* calculated from biological replicates across experimental sampling points (n = 4). (C) ATAC-seq signal within consensus ATAC-seq peaks was compared between all samples, using Spearman’s ρ to cluster samples. (D) Comparison of GO annotations associated with accessible promoters identified from each library. The degree of enrichment is indicated using normalized Z-scores.

Many ATAC-seq peaks, from both the LD and DD samples, were mapped within 1500 bp upstream to the TSS (S2 Fig), marking the accessible chromatin of extended promoters. ATAC-seq libraries (LD and DD) were enriched, on average, with 20.87% (SD ±1.76) promoter regions, 20.35% (SD ±1.9) intron regions, and 43.1% intergenic regions (Proximal– 16.6% SD ±1.05, Distal– 26.5% SD ±2.65) that may act as distant regulatory elements (Fig 2A and 2B). Comparing the DD and LD libraries revealed time based clustering, which shows that chromatin accessibility is maintained in *N*. *vectensis* under constant conditions (Fig 2C). Moreover, gene ontology (GO) enrichment analysis of genes with accessible promoters showed that nucleic acid binding and transcription regulation activities remain similar between the two light regimes. Interestingly, DD-specific accessible gene promoters were highly enriched with rhodopsin-like, G-protein-coupled receptors (GPCRs), which are related to external signal transduction [20] (Fig 2D).

### Chromatin accessibility at rhythmic genes

**Fig 3:**
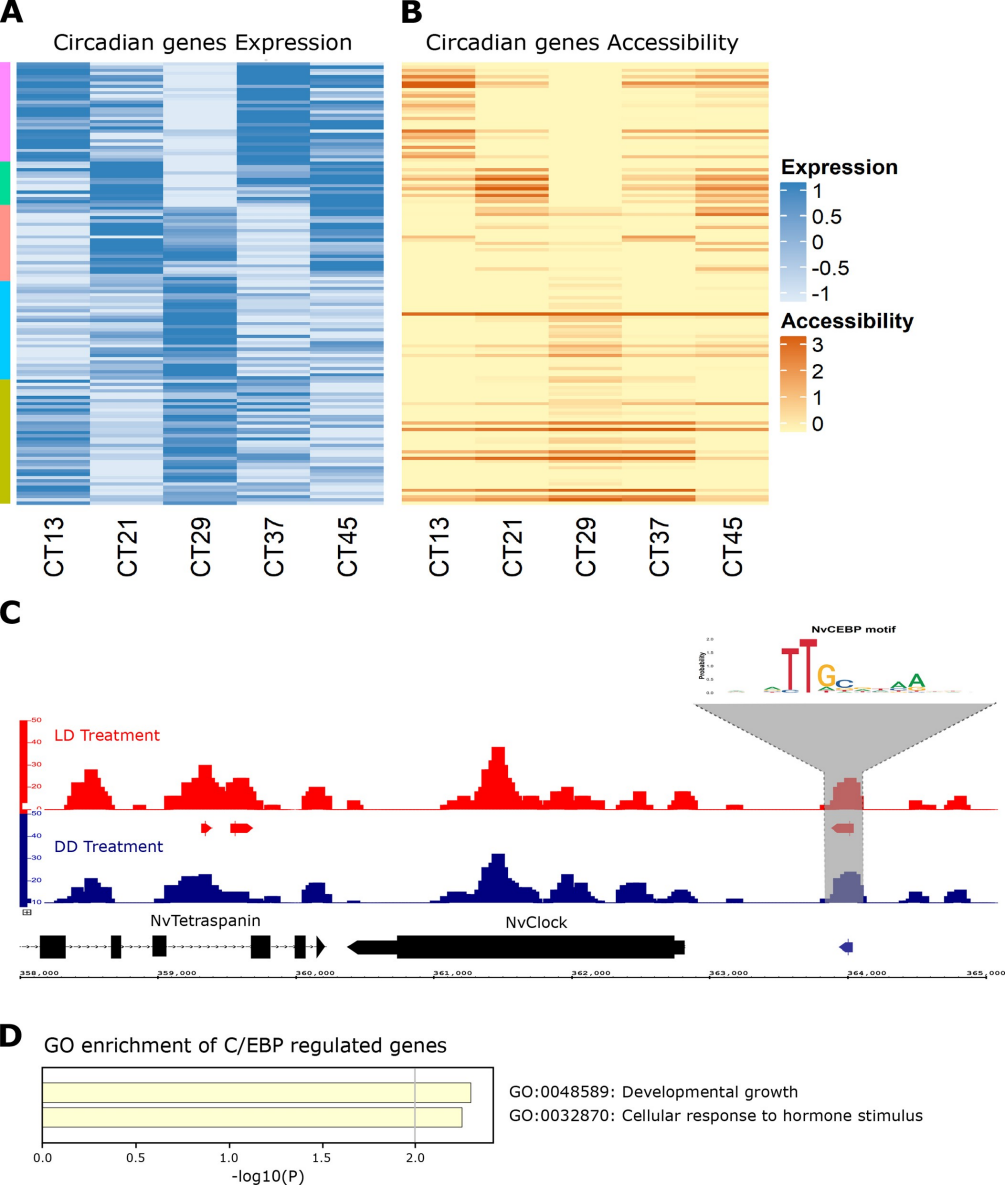
(A—B) 139 out of 180 genes that were found to be rhythmic in a previous RNA-seq experiment (Oren *et al*., [15]) clustered by 5 expression groups and aligned to their accessibility score. RNA expression score is in -log_2_(fold-change) colored in blue in the range of -1 to 1. ATAC-seq accessibility score is in -log_2_(FPKM), colored in orange in the range of 0 to 3. (C) Browser view of peaks from two treatments (LD CT13 –red, DD CT13 –blue). LOGO graph indicates the NvC/EBP motif found within marked peaks. Arrows under the peaks track indicate true peaks with IDR cut-off of 0.05. (D) Enriched terms across 15 rhythmic genes, regulated by NvC/EBP in their promoter.

Previously, we showed an association between circadian locomotor activity rhythm and transcriptional profile in *N*. *vectensis*. Using Fourier analysis, diel rhythmicity (i.e., 24-h periodicity) was identified in many genes. From these, 180 transcripts exhibiting significant oscillations (G-factor >0.5) were selected for further analysis. Through K-means clustering, these transcripts were divided into five clusters, each representing a different peak time of chronological expression (S1C Fig) [15]. To further study the relationship between promoter accessibility and gene expression, 139 genes were selected, with promoter regions (within 1500 bp upstream of TSS) showing higher accessibility at CT13 (the time point with the strongest change in gene expression between the five clusters) than the average promoter accessibility, genome-wide. To test the association between all the data, we conducted a Pearson correlation test, finding a significant correlation between expression and accessibility (R^2^ = 0.343 and P-value<0.01 –see S2 Table). Moreover, comparing accessibility and expression within each time point showed that promoters’ accessible sites oscillated during a 24h cycle and correlated to the syn-expression pattern of circadian genes, as shown in Fig 3A and 3B and S2 Table (Note: at CT 37, the correlation is not significant, although the overall pattern is visible). However, we have to remain skeptical, as not all genes in the presented list (S4 Table) correspond to the rule of accessibility and expression correlation. For example, a subset of genes that share a common TF site (CEB/P) show no significant correlation between expression and accessibility. These genes show relatively high accessibility throughout the experiment, with an average–log_2_(FPKM) rate of 2.5 and a SE of ±0.3. These genes are related to core clock mechanism and development, as we show later.

### Accessible sites containing motifs and binding sites of TFs

Selective activation of functional regulatory DNA elements defines where TFs may bind and act. Therefore, to predict the identity of active TFs in treatment-specific peaks, the enrichment of sequence motifs was computed using the HOMER motif analysis tool [21]. The identified motifs were divided into three groups: (i) common motifs—motifs that were enriched in both treatments, relative to their abundance within the genome (see Table 3), (ii) LD-enriched (see Table 4), and (iii) DD-enriched (see Table 5). Many of the identified motifs correspond to binding sites of TFs with previously-identified roles in regulating rhythmic processes [22,23]. For example, the *MYB* motif, enriched in LD-specific peaks, was shown to have an essential role in circadian rhythm maintenance in *Arabidopsis* [24]. Interestingly *MYB* motif was enriched around the promoters of differentially expressed (DE) genes under LD conditions, particularly within clusters 1, 2, and 5. Another example is the homeobox motifs, enriched in both the LD and DD treatments, as well as around genes from clusters 1, 3, 4 and 5. Homeobox factors, in particular members of the NK homeobox gene family, with motifs enriched in both treatments, have been shown to contribute to rhythmic regulatory processes in mammals [25]. Within cluster 5, 16 cyclic genes, including *NvClock* (a core component of the circadian clock machinery), contain the binding motif of C/EBP in their accessible promoter region (Fig 3C and S3 Table). C/EBP acts as an enhancer of promoter activation [26,27], and its association to *NvClock* promoter was predicted previously [17]. Functional analysis of these 16 genes reveals that they are related to the GO term’s developmental growth (GO:0048589) and cellular response to hormone stimulus (GO:0032870) (Fig 3D) [28]. These results should be treated with skeptical eyes, as it cannot be excluded that other, unidentified, TFs might be involved in circadian rhythm regulation in *N*. *vectensis*.

**Table 3:** Treatments common motifs.

Name	LD enrichment	LD fold-enrichment	P-value (LD)	DD enrichment	DD fold-enrichment	P-Value (DD)
YAP7	0.05	5.00	1.00E-03	1.91	10.61	1.00E-50
TaMYB80	1.39	2.01	1.00E-07	6.08	2.84	1.00E-44
CEBP:AP1(bZIP)	0.05	5.00	1.00E-03	1.98	5.66	1.00E-32
YAP1	3.58	1.56	1.00E-08	1.91	3.75	1.00E-20
GATA15	0.37	9.25	1.00E-12	2.31	3.08	1.00E-19
Pax7	0.62	12.40	1.00E-26	0.4	2.86	1.00E-14
Mef2a	4.44	11.00	1.00E-36	6.58	1.60	1.00E-12
ERE	1.75	1.41	1.00E-03	2.03	1.71	1.00E-05
IRF4	3.71	1.28	1.00E-03	4.42	1.39	1.00E-04
Nkx3-1	13	13.00	1.00E-07	1.11	1.11	1.00E-03

**Table 4:** LD enriched motifs.

Name	% of Targets	% of Background	P-value	log P-pvalue
Mef2a	0.99%	0.09%	1.00E-36	-8.34E+01
tll	1.57%	0.37%	1.00E-27	-6.27E+01
Pax7	0.62%	0.05%	1.00E-26	-6.05E+01
br-Z3	1.20%	0.23%	1.00E-25	-5.82E+01
Six1	0.66%	0.07%	1.00E-21	-4.98E+01
SWI4	1.24%	0.32%	1.00E-19	-4.50E+01
br-Z2	0.33%	0.01%	1.00E-19	-4.49E+01
HMG-1	0.22%	0.01%	1.00E-15	-3.65E+01
MZF1	2.52%	1.18%	1.00E-15	-3.48E+01
Unknown-ESC-element	0.20%	0.01%	1.00E-14	-3.26E+01
SWI4	0.22%	0.01%	1.00E-13	-3.17E+01
Nr2f6	1.20%	0.42%	1.00E-12	-2.96E+01
GATA15	0.37%	0.04%	1.00E-12	-2.77E+01
OLIG2	0.15%	0.00%	1.00E-11	-2.71E+01
FHL1	0.16%	0.01%	1.00E-10	-2.52E+01
HSF1	0.22%	0.02%	1.00E-10	-2.38E+01
ZNF410	0.13%	0.00%	1.00E-09	-2.30E+01
bZIP910	0.16%	0.01%	1.00E-09	-2.17E+01
PRDM14	1.13%	0.47%	1.00E-09	-2.09E+01
YAP1	3.58%	2.30%	1.00E-08	-1.97E+01
gt	1.11%	0.48%	1.00E-08	-1.91E+01
ROX1	2.21%	1.27%	1.00E-08	-1.85E+01
Nkx3-1	0.13%	0.01%	1.00E-07	-1.82E+01
Cf2-II	0.40%	0.09%	1.00E-07	-1.82E+01
MYB3	0.89%	0.36%	1.00E-07	-1.79E+01
TaMYB80	1.39%	0.69%	1.00E-07	-1.73E+01
XBP1	0.15%	0.01%	1.00E-07	-1.64E+01
NAC058	0.09%	0.00%	1.00E-06	-1.51E+01
MYB.PH3(2)	0.15%	0.01%	1.00E-06	-1.47E+01
br(var.4)	0.09%	0.01%	1.00E-05	-1.18E+01
Tcf1_2	0.11%	0.01%	1.00E-04	-1.10E+01
Trl	1.10%	0.61%	1.00E-04	-1.08E+01
HMRA2	0.09%	0.01%	1.00E-03	-8.52E+00
hkb	0.05%	0.00%	1.00E-03	-8.03E+00
YAP7	0.05%	0.00%	1.00E-03	-8.03E+00
CEBP:AP1(bZIP)	0.05%	0.00%	1.00E-03	-8.03E+00
AP2	4.87%	3.85%	1.00E-04	-9.35E+00
IRF4	3.71%	2.90%	1.00E-03	-8.02E+00
ERE	1.75%	1.24%	1.00E-03	-7.31E+00

**Table 5:** DD enriched motifs.

Name	% of Targets	% of Background	P-value	log P-pvalue
YER130C	8.11%	2.08%	1.00E-91	-2.11E+02
Dof2	4.29%	0.55%	1.00E-91	-2.10E+02
Rhox11	5.02%	0.86%	1.00E-84	-1.95E+02
OPI1	5.70%	1.31%	1.00E-72	-1.66E+02
unc-86	2.29%	0.16%	1.00E-69	-1.61E+02
RUNX2	1.83%	0.09%	1.00E-67	-1.56E+02
ETV2	7.81%	2.48%	1.00E-66	-1.54E+02
Arid5a	2.06%	0.13%	1.00E-66	-1.54E+02
br-Z1	2.71%	0.29%	1.00E-65	-1.51E+02
Mef2c(MADS)	3.39%	0.52%	1.00E-62	-1.44E+02
vnd	1.38%	0.04%	1.00E-62	-1.44E+02
SFP1	2.69%	0.32%	1.00E-59	-1.37E+02
Gfi1b	1.96%	0.15%	1.00E-57	-1.33E+02
fkh	3.67%	0.68%	1.00E-57	-1.33E+02
MGA1	1.28%	0.04%	1.00E-57	-1.32E+02
nub	2.49%	0.31%	1.00E-54	-1.25E+02
YAP7	1.91%	0.18%	1.00E-50	-1.16E+02
HMRA1	2.49%	0.35%	1.00E-49	-1.14E+02
Dfd	1.21%	0.05%	1.00E-49	-1.14E+02
ZNF354C	0.83%	0.01%	1.00E-46	-1.06E+02
Irx2	1.00%	0.03%	1.00E-45	-1.05E+02
TaMYB80	6.08%	2.14%	1.00E-44	-1.03E+02
Pou6f1_1	3.37%	0.77%	1.00E-43	-9.93E+01
WRKY43	3.82%	1.06%	1.00E-39	-9.03E+01
YER130C	7.13%	3.10%	1.00E-35	-8.25E+01
ZNF264(Zf)	15.60%	9.41%	1.00E-34	-7.92E+01
CEBP	1.98%	0.35%	1.00E-32	-7.52E+01
Sox1	0.83%	0.04%	1.00E-31	-7.25E+01
zen	2.71%	0.71%	1.00E-29	-6.89E+01
Hic1	1.91%	0.38%	1.00E-28	-6.54E+01
Deaf1	6.15%	2.83%	1.00E-27	-6.32E+01
SIP4	0.63%	0.03%	1.00E-22	-5.18E+01
YAP1	1.91%	0.51%	1.00E-20	-4.73E+01
GATA15	2.31%	0.75%	1.00E-19	-4.45E+01
Rara	2.46%	0.84%	1.00E-19	-4.39E+01
Nr2e3	3.37%	1.38%	1.00E-19	-4.38E+01
Dof2	3.97%	1.81%	1.00E-18	-4.20E+01
Nr5a2	2.03%	0.73%	1.00E-14	-3.43E+01
Pax7	1.86%	0.65%	1.00E-14	-3.25E+01
GIS1	6.40%	3.89%	1.00E-13	-3.13E+01
Mef2a	6.58%	4.10%	1.00E-12	-2.93E+01
ZNF136	1.38%	0.56%	1.00E-08	-1.93E+01
RLR1	5.88%	4.12%	1.00E-07	-1.62E+01
Nkx2-2	17.10%	14.35%	1.00E-06	-1.41E+01
RUNX-AML	7.33%	5.53%	1.00E-05	-1.38E+01
FOXM1	12.61%	10.27%	1.00E-05	-1.35E+01
Nkx2-5	19.66%	16.89%	1.00E-05	-1.29E+01
ERE(NR),IR3	2.03%	1.19%	1.00E-05	-1.21E+01
IRF4	4.42%	3.17%	1.00E-04	-1.13E+01
MafK	1.51%	0.83%	1.00E-04	-1.11E+01
ZBTB18(Zf)	3.06%	2.08%	1.00E-04	-1.05E+01
MafA	6.20%	4.78%	1.00E-04	-1.04E+01
Foxo1	17.48%	15.30%	1.00E-04	-9.29E+00
FOXP1	6.65%	5.32%	1.00E-03	-8.72E+00
Nkx3-1	22.35%	20.15%	1.00E-03	-8.03E+00
NF-E2	0.95%	0.51%	1.00E-03	-8.03E+00
Myf5	4.42%	3.41%	1.00E-03	-7.81E+00
FXR(NR),IR1	2.64%	1.91%	1.00E-03	-7.13E+00

### ATAC-seq accessibility and RNA-seq expression patterns

**Fig 4:**
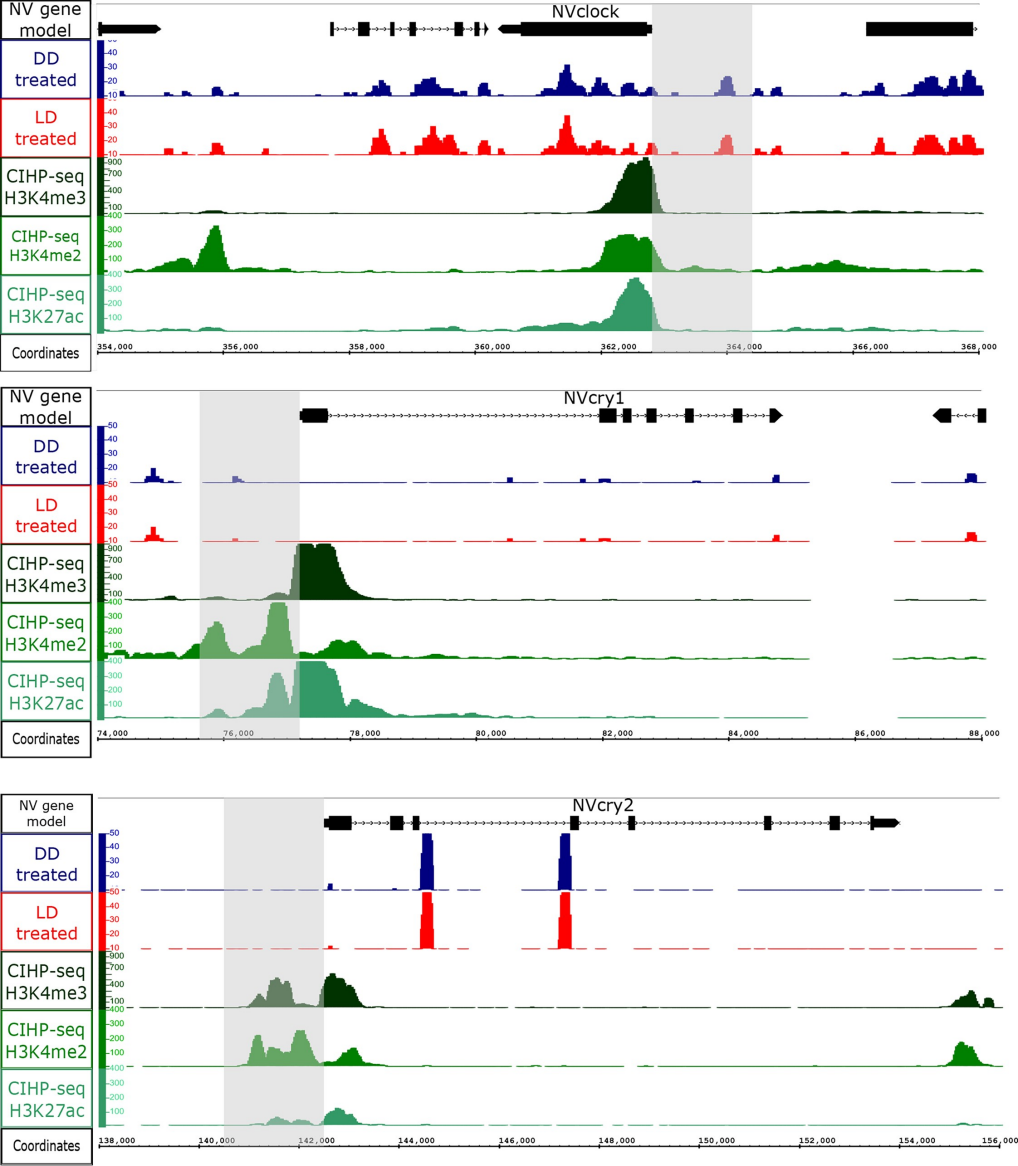
The high expression period of *NvClock* overlaps with the ATAC-seq time point CT13, and peaks occur within the *NvClock* promoter (gray rectangle). In contrast, the high expression periods of *NvCry1* and *NvCry2* do not overlap the ATAC-seq sampling time point, and no ATAC-seq peaks occur within their promotors (grey rectangles). ATAC-seq LD peaks in red, ATAC-seq DD peaks in blue, compared to Chip-seq published data peaks [32]–H3K4me3 in dark green, H3K4me2 in green and H3K27ac in turquoise.

Promoter accessibility is essential for gene expression, so the proportion of promoters in the accessible chromatin loci is non-random. Many expression patterns found in our ATAC-seq data were visible in the RNA-seq data as well. For example, *NvClock* exhibits an expression peak from late day to early night (CT9-CT13), which overlaps our ATAC-seq results from CT13 that shows its promoter to be accessible (Fig 3C). In contrast, two cryptochromes (key components of the biological clock mechanism), *NvCry1* and *NvCry2*, exhibit transcriptional rhythms, with peak expression during the day (CT4-CT11 for *NvCry1* and CT0-CT4 for *NvCry2*) [15], prior to the ATAC-seq sampling at CT13. Concordantly, we did not find their promoters to be accessible in either treatment. Overall, the patterns observed for *NvClock*, *NvCry1*, and *NvCry2* are aligned with the correlation between chromatin accessibility measured by ATAC-seq and expression profiles measured by RNA-seq (Fig 4 and S4 Table). Furthermore, the RNA data identified four minicollagen genes with strong rhythmicity, while three of these four gene promoters were identified as significantly accessible at CT13 (p-value < 0.05). Minicollagen is an important feature of the nematocyst structure and is expressed from the early stages of nematocyst morphogenesis until capsule maturation [29]. By identifying enriched TF motifs within the peak sequences, potential gene regulators can be revealed. Within the peaks, at these gene promoters, we have identified motifs for *C/EBP*, *Sox1*, *Pax-4*, and *Pax-6*, all of which have been shown to act as clock-controlled gene regulators in mammals [27].

### ATAC-seq identifies distal regulatory regions in adult *Nematostella vectensis*

Enhancers are distinct genomic regions containing binding site sequences for TFs that can regulate the transcription of a target gene. Along the linear genomic DNA sequence, active enhancers, marked with H3K27ac histon modification marker, can be located at a great distance from their target genes. About 70% of H3K27ac-marked enhancers in mammals are active and positively affect transcription *in vivo* [30,31]. Comparing our ATAC-seq profiles with previously published H3K27ac ChIP-seq [32] revealed that ~50% of ATAC-seq peaks overlap with H3K27ac sites in both treatments (S5 Table), indicating their potential enhancer activity. Out of ~5000 previously ChIP-seq-predicted enhancer elements in *N*. *vectensis* during different early development stages, our analysis identified 259 LD-treated and 333 DD-treated enhancers that overlap with the H3K27ac histone mark, and a total of 174 enhancers shared between the two treatments (S3 Fig).

## Discussion

The ATAC-seq technology was applied using a small quantity of nuclei, proved to be effective and produced ATAC-seq libraries with low, non-nucleic DNA contamination (less than 5% mitochondrial DNA per sample) and median depth of 10 million high-quality unique reads that represent the accessible chromatin of *N*. *vectensis* under the different treatments. Moreover, ATAC-seq can be utilized to increase the proportion of regulatory genomic features, such as promoters to ~20% within sequenced libraries. Therefore, ATAC-seq can act as a powerful tool in the *N*. *vectensis* genome research, and in other cnidarians, and can be applied to study epigenetics downstream to DNA methylation, RNA-seq and more.

In this work, we aim at illuminating the landscape of accessible chromatin, within the *N*. *vectensis* genome, to uncover valuable information about the active CREs and the TFs that bind them, and further enable us to examine the relationship between CREs and gene expression. Among the two light regimens surveyed, 8366–36582 peaks with IDR cut-off of 0.05 were identified, enabling the prediction of TFs binding sites within the accessible genome, specifically around rhythmic genes. The overlaps of ATAC-seq libraries with rhythmic genes lead us to conclude that there is an association between gene expression and DNA accessibility in *N*. *vectensis*, as presented in our time point sampling.

To identify groups of genes based on promoter accessibility, we conducted a GO enrichment analysis, comparing genes with accessible promoters to the full *N*. *vectensis* gene set. Our results showed a strong enrichment of rhodopsin-like GPCRs, particularly in the DD treatment. The rhodopsin-like GPCRs represent a diverse protein family that includes hormone receptors, neurotransmitters and photoreceptors, all of which transduce extracellular signals through interactions with nucleotide-binding proteins [33]. Remarkably, the rhodopsin-like GPCRs in the DD-treated samples were enriched with SOX gene family binding sites, relative to LD-treated samples. The SOX TF family is found throughout the animal kingdom and is important in a variety of homeostasis and regeneration contexts. Most of the SOX genes found in mammals have homologs in invertebrates, including non-bilaterian lineages such as sponges [34,35]. There is also a direct connection between the SOX family and the circadian clock, as many SOX genes have been shown to be clock-controlled [36]. the increase in SOX binding sites in DD-treated samples and not in LD-treated samples can be explained by the loss of coupling between the biological clock and the cell cycle [37].

We found that gene expression rhythmicity corresponded with changes in DNA accessibility within promoters of 139 out of 180 previously-identified rhythmic genes. However, this general rule does not apply to all circadian genes, and there was a subset of genes that showed relatively high and continues accessibility rate throughout the experiment (S3 Table and S4 Table). This state of constantly accessible genes suggests that the expression rhythmicity has more than one general regulatory mechanism controlling this process [38]. When rhythmic genes were sorted according to their expression patterns, the enriched motifs within each cluster revealed a complex picture of regulatory activity. Analysis of accessible regions of individual promoters enabled predictions of TFs that are likely to bind to and regulate the associated genes. For example, the identification of a C/EBP motif within a peak on the *NvClock* promoter (Fig 3C) is important as C/EBP association to *NvClock* promoter was previously predicted [17]. Moreover, C/EBP is overrepresented in promoters of clock-controlled mammal genes [26] and acts as an enhancer of promoter activation. The C/EBP motif is found in 15 more rhythmic gene promoters theat are characterized in this work (S3 Table). Another interesting finding is the presence (or absence) of homeobox-related TFs in rhythmic genes regulation. The identified motifs were enriched with NK Homeobox and HOX family motifs–some of which contribute to rhythmic regulatory processes [25] including *CUX1*, *LIN-39* (*HOX3A*), *caudal* and *CDX2* (Caudal-Type Homeobox 2). Interestingly, *caudal* has also been identified (in gene cluster 3) and previously reported as a synchronizer of locomotor activity in crayfish [39]. Our observation that different motifs are more enriched in LD and not in DD, or vice versa, indicates that the light regimen affects the regulatory network that is activated around rhythmic genes. To investigate this issue, further work needs to be conducted, including a high-resolution sampling of these genes’ regulatory landscapes, to elucidate if these changes are linked to the rhythmic cycle or due to light deprivation impact. Surprisingly, many of the 41 rhythmic genes that do not have accessible promoters exhibited peak transcript expression at other times of day. For example, *NvCry* genes have expression peaks that did not overlap with the CT13 sampling point; (*NvCry1* expression peak is at CT4-CT11 and *NvCry2* expression peak is at CT0-CT4). As cryptochromes are photoreceptors, their expression profile peaks at mid-day (light time) [12,15], it is not surprising that at CT13 (dark time) we did not observe accessibility nor expression. Nonetheless, *NVcry1* becoms accessible at sampling point CT29, This could indicate that its proximate chromatin region is more packed or inhibited, and further investigation is needed to validate this conclusion.

The genomic sequence in the immediate vicinity of the TSS, which is also known as the core promoter, is sufficient to assemble the Pol II complex with its associated proteins. However, transcription is often weak in the absence of regulatory DNA regions, such as enhancers, that are more distant from the TSS. Enhancers are key regulators of temporal and tissue-specific gene expression that display important and conserved functions and can be found at thousands of base pairs upstream or downstream to their target promoters [40]. By comparing ATAC-seq to a known list of enhancers found in *N*. *vectensis*, we could identify enhancers that can serve as potential targets for rhythmic gene regulation (see S3 Fig).

Finally, the work presented here shows the association between gene expression and DNA accessibility by integrating two sequencing methods. This improves our understanding of the *N*. *vectensis* regulatory landscape, exposing the regulatory elements that participate in gene regulation genome-wide, which can be important for chronobiology and evolutionary investigations and future studies in epigenetics.

## Materials and methods

### Animal culture

Adult *N*. *vectensis* were kept in a plastic container filled with two liters of artificial seawater at a salinity of 12 PSU (*Nematostella* medium), under natural light and at a constant temperature of 18°C. Between 50 and 100 individuals were kept in each container in a recirculating water system. Animals were fed 5 times a week with freshly hatched brine shrimp (*Artemia* nauplii).

### Experimental design

Female *N*. *vectensis* were incubated under two different light regimens: LD (12h light:12h dark) or DD (constant darkness), for 45 hours at a constant temperature of 18°C. Biological duplicates were sampled every 8 hours at CT13, CT21, CT29, CT37 and CT45 from both conditions and were processed as described in the “ATAC-seq nuclear isolation and library preparation” section. We did not sequence sample DD CT29 due to technical problems. These time points were chosen based on gene enrichment data from RNA-seq experiments previously published. The ATAC-seq sampling interval was due to the timing requirements of the ATAC-seq protocol (approximately 6–7 hours).

### ATAC-seq nuclear isolation and library preparation

Nuclei were isolated from adult *Nematostella* that were incubated in different lighting treatments. From each sample, tissue was suspended in 500 μL PBS-NAC 2% (N-acetyl-cysteine, sigma) by pipetting in a 1.5 mL tube [41]. The suspension was centrifuged at 1500 xg for 5 minutes, at 4°C. The pellet was re-suspended in 500 μL PBS and cells were counted. 400,000 cells were then re-suspended in 500 μL PBS and centrifuged at 1500 xg for 5 minutes, at 4°C. The pellet was suspended in 50 μL of ATAC-seq lysis buffer (10mM TRIS-Cl pH 7.4, 10mM NaCl, 3mM MgCl2, 0.1% IGEPAL CA630) and centrifuged at 300 xg for 10 minutes, at 4°C. The supernatant was collected and kept in a 1.5 mL tube on ice. The pellet was re-suspended in 50 μL and centrifuged at 300 xg for 10 minutes, at 4°C. The supernatant was combined with the supernatant from the previous step. Then 9 μL of isolated nuclei were stained with DAPI to verify the isolation of intact nuclei. The isolated nuclei were then centrifuged at 1500 xg for 10 minutes, at 4°C. Immediately following this centrifuge step, the pellet was re-suspended in the transposase reaction mix (25 μL 2× TD buffer, 2.5 μL transposase (Illumina REF: 15028212) and 22.5 μL nuclease-free water). The transposition reaction was carried out for 30 minutes, at 37°C. Directly following transposition, the sample was purified using an Invitrogen PureLink PCR purification kit (REF: K310001). Following purification, library fragments were amplified using 1× NEBnext PCR master mix (#M0541S) and 1.25 μM of custom Nextera PCR primers, forward and reverse, using the following PCR conditions: 72°C for 5 minutes, 98°C for 30 seconds and a variable number of cycles as needed (we added 4–9 cycles) at 98°C for 10 seconds, 63°C for 30 seconds and 72°C for 1 minute. To reduce GC and size bias in our PCR, we monitored the PCR reactions using qPCR to stop amplification before saturation. To do this, we amplified the full libraries for 5 cycles, after which we took a 4-μl aliquot of the PCR reaction and added 6 μl of the PCR cocktail with Sybr Green (Promega, REF: A6001), at a final concentration of 0.6×. We ran this reaction for 20 cycles to determine the additional number of cycles needed for the remaining 46-μl reaction. The libraries were purified using Agencourt AMPure XP beads (cat. No. 63881) and analyzed on a TapeStation. Primers used to amplify ATAC-seq libraries see S6 Table (Note: Ad1_noMX is a global primer used in all libraries).

### Data analysis

Samples of whole *N*. *vectensis* were prepared using single-end 50bp reads from a single Illumina HiSeq run. Treatments were run on one lane of Illumina HiSeq2000. On average, ~50 million single-end reads were obtained for each sample.

Sequenced reads were aligned to the Nemve1 *Nematostella vectensis* genome using bowtie [42]. Only unique mapped reads were used. Peaks were called by applying MACS2 [43] with the following parameters: -g 450000000—nomodel—extsize 75—shift -30. TF-binding motifs enrichment were identified within the peaks using scripts within HOMER [21]: findMotifsGenome.pl and annotatePeaks.pl were used with default parameters and the Nemve1 genome was used as background [44].

To compare chromatin accessibility with circadian patterns in gene expression, we evaluated a set of 180 transcripts that were previously shown to exhibit a diel expression pattern in *N*.*vectensis* [15]. The genes had been sorted into 5 clusters with similar temporal expression patterns, using a K-means clustering, implemented in MatLab as described by Oren *et al*., [45]. Pearson correlation tests were apllied to assess correlation between gene expression and DNA accessibility. We have compared all genes’ expressions to promoter accessibility within a single time point (for example, gene expression at CT13 was compared to promoter accessibility at CT13). In addition, we compared the total RNA-seq data from our 139 gene list to the total ATAC-seq data at all time points (see S2 Table). To identify possible distal enhancer sites in the ATAC-seq data, ChIP-seq data was used from a previous study of histone markers in *N*.*vectensis* [32]. Reads were downloaded from NCBI GEO (accession number: GSE46488) and aligned to the genome using bowtie2. Only uniquely mapped reads were used. Peaks were called by applying MACS2 [43] with default parameters. Further analysis was performed using the BamTools and BEDTools suites [46,47]. Gene promoters found within treatment specific peaks were defined and subsequently, analyzed for enriched GO terms using the metascape suit [28].

## Supporting information

S1 Fig (A) *Nematostella vectensis* locomotor activity under a 12-hr light: 12-hr dark cycle (LD). (B) *Nematostella vectensis* locomotor activity under constant dark (DD). Yellow indicates light hours, grey indicates dark hours and pale blue indicates dark during subjective day. (C) 139 out of 180 genes that were found to be rhythmic in a previous RNA-seq experiment, clustered by expression groups. Above the heatmap is the aligned distance moved, as measured in LD. Data from *Oren et al*., [15].
(EPS)

S2 Fig Histograms from all time points, sampled from LD and DD, showing the peak distribution around TSS. The red bars indicate the approximate place of the nearest next TSS (~8,600bp).
(EPS)

S3 Fig (A-B) We determined the overlap of ATAC-seq peaks with H3K27ac ChIP-seq peaks in LD-treated and DD-treated *N*.*vectensis*. (C-D) We determined the overlap of ATAC-seq peaks with H3K4me2 peaks in LD-treated and DD-treated *N*.*vectensis*. (E-F) We determined the overlap of ATAC-seq peaks with H3K4me3 peaks in LD-treated and DD-treated *N*.*vectensis*. (G) Comparison of H3K27ac peak position to Planula and Gastrula enhancer list from previously published Chip-seq data (*Schwaiger et al*., [32]).
(EPS)

S1 Table Correlation comparison between biological replicates of ATAC-seq libraries reads after filtering PCR duplicates. P-value < 0.01 for all results.
(XLSX)

S2 Table Pearson R^2^ correlation of gene expression measured in–log_2_(fold-change) and promoter accessibility measures in–log_2_(FPKM) of 139 rhythmic genes. (XLSX)

S3 Table List of 16 rhythmic genes, and their annotations, found to have a C/EBP accessible motif within their promoter region. (XLSX)

S4 Table Annotation, expression and accessibility patterns of 139 Nematostella transcripts exhibiting circadian-like periodicity in expression. (XLSX)

S5 Table Pie charts of genomic annotations showing histone acetylation marks identified from a previous ChIP-seq study, performed on adult female *Nematostella* by *Schwaiger et al*., [32] (left column). Center and right columns show genomic annotations of ATAC-seq peaks that overlap with the ChIP-seq data. Promoters-TSS: -1500 bp to TSS, proximal Intergenic: -5000 to -1501 or TTS to 5000bp.
(EPS)

S6 Table A list of all primers used to prepare ATAC-seq libraries. (XLSX)
